# Distribution and prevalence of ticks and tick-borne disease on sheep and cattle farms in Great Britain

**DOI:** 10.1186/s13071-020-04287-9

**Published:** 2020-08-10

**Authors:** Katie Lihou, Hannah Rose Vineer, Richard Wall

**Affiliations:** 1 School of Biological Sciences, University of Bristol, Bristol, BS8 1TQ UK; 2 Department of Infection and Microbiome, Institute of Infection, Veterinary and Ecological Sciences, University of Liverpool, Liverpool, UK

**Keywords:** Disease risk, Livestock, Pathogen, *Ixodes ricinus*, TBD management

## Abstract

**Introduction:**

The most abundant and widespread tick species in Great Britain, *Ixodes ricinus*, is responsible for the transmission of a range of pathogens that cause disease in livestock. Empirical data on tick distribution and prevalence are required to inform farm management strategies. However, such data are largely unavailable; previous surveys have been rare and are usually relatively localised.

**Methods:**

A retrospective questionnaire survey of farmers was used to assess the reported prevalence of ticks on livestock across Great Britain. Spatial scan statistics and kernel density maps were used to assess spatial clustering and identify areas of significantly elevated risk, independent of the underlying distribution of respondents. Logistic regression models were used to identify risk factors for tick presence.

**Results:**

Tick infection risk to livestock is shown to be spatially aggregated, with areas of significantly elevated risk in north Wales, northwest England and western Scotland. Overall, the prevalence of farms reporting tick presence was 13% for sheep farms and 6% for cattle farms, but in “hot spot” clusters prevalence ranged between 48–100%. The prevalence of farms reporting tick-borne disease overall was 6% for sheep and 2% for cattle, but on farms reporting ticks, prevalence was 44% and 33% for sheep and cattle farms, respectively. Upland farming, larger flock sizes, region and the presence of sheep on cattle farms were all significant risk factors for tick presence.

**Conclusions:**

These data have important implications for assessing both the risk of tick-borne disease in livestock and optimising approaches to disease management. In particular, the study highlights the need for effective livestock tick control in upland regions and the southwest, and provides evidence for the importance of sheep as tick maintenance hosts. 
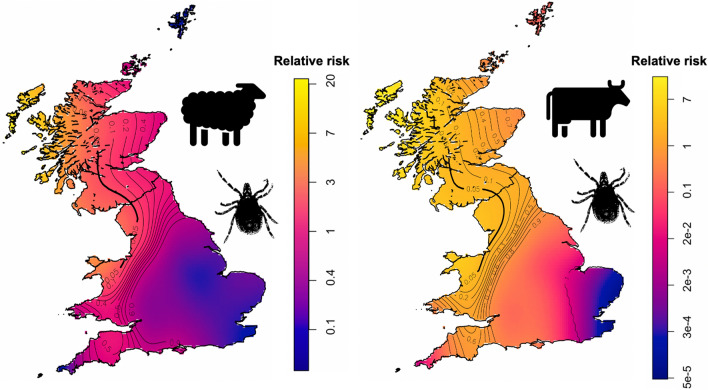

## Background

In livestock husbandry, ticks are important both as direct blood-feeding parasites and as vectors of a range of production-limiting pathogens with economic and welfare impacts on the livestock industry through reduced production and animal mortality [1, 2]. The most widespread tick vector of livestock pathogens in northern Europe is *Ixodes ricinus* [3, 4], with clinical cases occurring during the periods of tick activity, primarily from the spring through to autumn. Predicting the distribution and incidence of tick-borne disease (TBD) can be complex, since it depends on both the availability of hosts and abundance of questing ticks, which varies across seasons, years and regions [5], reflecting variations in local microclimate and habitat [6]. However, it is also affected by the prevalence of pathogens within co-occurring transmission hosts [7] and the immunity generated by prior exposure [8, 9].

While *I. ricinus* is widespread in the UK, populations are highest in areas where the habitat, microclimate and host availability are conducive to high survival [10]. These are generally areas of rough grassland, heath, moorland and woodland with a moist vegetation layer, where the relative humidity remains above the critical value of 80%, required to prevent desiccation [3, 10]. These areas often have high populations of wild hosts, such as rabbits, deer or ground nesting birds and are unsuitable for crops, so can support only extensive livestock grazing [3, 11]. Sheep in particular are thought to be one of the most important host species for all *I. ricinus* life-cycle stages in pasture or moorland [12]. Control is difficult as *I. ricinus* is generally non-host specific, infecting a variety of mammals and birds and spending the majority of its life-cycle off-host in the environment [13].

For sheep in the UK, ticks are particularly important in the transmission of tick-borne fever (anaplasmosis), louping-ill virus (LIV) and tick pyaemia. Anaplasmosis is a widely dispersed disease throughout Europe and can be major problem in livestock production, affecting both sheep and cattle [14]. The bacterium, a ruminant-specific variant of *Anaplasma phagocytophilum*, infects granulocytes, which can result in secondary infections due to immunosuppression [15]. Transstadial transmission of *A. phagocytophilum* can occur, whereby the pathogen is transmitted from one tick developmental stage to the next. Louping-ill virus (LIV), also called infectious ovine encephalomyelitis, can also be transmitted by transstadial transmission. LIV is an acute viral disease which affects the brain and the nervous system caused by a flavivirus closely related to the causal agent of tick-borne encephalitis. Louping-ill has been reported from most regions in the north and west of the UK, but has not been found in central or east England [16]. Louping-ill has also been identified in Ireland and regions of France and Norway [17]. The disease is characterised by nasal discharge, fever, depression, ataxia, paralysis and coma, leading in many cases to death; morbidity in lambs can be up to 50% [18]. However, following early infection, lifelong immunity is sustained. Pyaemia results from the infection of lambs or sheep with S*taphylococcus aureus.* It is not directly transmitted by ticks, but *S. aureus,* usually found on the skin, may become pathogenic when transferred mechanically to the bloodstream *via* the bite of a tick. There is a strong association between tick-borne fever and pyaemia [19]. Pyaemia affects lambs born on, or newly introduced to, a tick infected area, and shows a peak in spring when tick abundance is high.

Regarding cattle in the UK, *I. ricinus* also transmits *A. phagocytophilum* and LIV, but importantly in some areas it is also a vector of *Babesia divergens*, the causal agent of redwater [2]. In the process of asexual division, intraerythrocytic *Babesia* cause lysis of erythrocytes, leading to haemoglobinaemia, haemoglobinuria and fever. In naïve adult hosts, infection may cause death within a few days. Milder forms of the disease, associated with juvenile or immune hosts, are characterized by fever and inappetence for a period of several days. In addition to transstadial transmission, *Babesia* is also transmitted *via* transovarial transmission within the tick, allowing the larvae, nymphs and adults of the next generation to transmit infection to cattle [2].

Despite the known range of tick-borne pathogens and concern over their impact on the welfare of livestock, there is very little quantitative information available about the prevalence of tick-borne disease in many areas of the UK. Previous systematic surveys in the UK have most usually been undertaken in the context of public health [20, 21], companion animal health [22–24], game birds [25], or by measuring tick abundance in the environment [26], which is not necessarily a good proxy for tick attachment risk [27]. Those studies of tick prevalence on livestock in the UK that have been undertaken, have usually been focussed on localised geographical regions with little area-wide context [28, 29]. Variability in sampling approach, time and context, also make reliable comparison between studies difficult. Furthermore, the fact that relatively few acaricidal pharmaceutical products are available with a label claim for efficacy against ticks in livestock, indicate that the control of ticks and TBD represents something of a neglected issue.

Appropriate strategies for tick and TBD management require an assessment of risk [30] and this necessitates up-to-date data on tick prevalence and distribution [31] in relation to livestock hosts. The aim of the work reported here, therefore, was to investigate the prevalence and spatial distribution of ticks and tick-borne disease reported in cattle and sheep in Great Britain and then to identify areas of elevated risk of tick attachment to livestock using spatial distribution modelling.

## Methods

### Questionnaire survey

A two-page retrospective postal questionnaire survey was sent to sheep and cattle farmers in Great Britain. The sample area was first stratified into 6 regions: Scotland, Wales, north, central, southwest and eastern England. A total of 7200 questionnaires were sent to a randomised selection of farms in each region sourced from a commercial database [32]. Questionnaires were only sent to farms meeting the following criteria: more than 50 sheep, or more than 20 beef cattle, or more than 30 dairy cattle, to avoid surveying smallholdings and ‘hobby farmers’, which may not be representative of commercial farms. Power analysis was used to obtain regional sample sizes to accurately estimate the proportion of cases in each region. The number of questionnaires sent out in each region, was based on the number of cattle or sheep holdings in each [33, 34], an estimated prevalence rate of 15%, an estimated response rate of 30% (based on previous farm-based survey studies, e.g. [35, 36], a confidence level of 95% and a margin of error of 5% (Win Episcope v.2.0; [37]). The questionnaire was sent out in November of 2018, and asked for general information about the holding and information about livestock numbers, tick presence and cases of TBD in the previous 12 months between November 2017 and October 2018, to control for temporal differences in tick abundance and distribution. The questionnaire contained separate sections for sheep and for cattle (see Additional file 1: Text S1).

The distribution of respondents was externally validated by qualitative comparison with the distribution of cattle and sheep holdings in the UK [38, 39]. Farm characteristics of respondents were externally validated by qualitative comparison with the ratio of dairy to beef farms [34] and the ratio of upland to lowland farms [40]. Questionnaires were also checked for internal consistency by removing questionnaires with missing tick presence/absence data and by qualitatively comparing monthly reported tick prevalence with expected temporal trends.

### Prevalence analysis

Responses for sheep or cattle farms were analysed separately, except when a direct comparison was made between sheep and cattle in reported tick prevalence. Differences in tick prevalence (proportion of farms reporting tick presence compared to tick absence) between regions and between farm terrain types (upland/lowland) were tested using Chi-square in R (version 3.6.1; [41]) using the *chisq.test* function. If expected values were less than five, Monte Carlo simulated *P*-values were used [42]. All prevalence values are reported ± their 95% confidence intervals.

### Spatial analysis

Farm postcodes were used for spatial analysis of cases (reported tick presence) and controls (reported tick absence) and converted to latitude and longitude [43]. Deviation from complete spatial randomness (CSR) was assessed by plotting significance envelopes of the G function, based on Monte Carlo simulation (100 repeats; *Gest* function in the *spatstat* R package (v.1.60–1; [44]). To identify case “hot spots” (areas which contain a higher density of points than would be expected with CSR) whilst accounting for the underlying distribution of the data points, the spatial relative risk of a respondent reporting the presence of ticks was estimated from the relative densities of cases and controls using the *risk* function in the *sparr* R package (v.2.2–13; [45]). An adaptive bandwidth was used, to compensate for potential over-smoothing in dense areas, calculated symmetrically with respect to cases and controls [45]. Diggleʼs edge correction was applied [45]. Asymptotic tolerance contours of *P*-values were plotted to show statistically significant areas of elevated risk (*tol.contour* function in the *sparr* R package; [46]).

Spatial clustering was assessed on different spatial scales using envelopes of the L-function (a standardised version of the K-function), which calculates the number of data points within a specified radius of each point (*Lest* function in the *spatstat* R package). L-functions were compared between case and control points to detect whether case points were more clustered than clustering caused by the underlying point distribution. Clustering was assessed for significance using SaTScan^TM^ [47], which uses Monte Carlo discrete spatial scan statistics to detect non-random clusters of cases, whilst adjusting for the underlying spatial distribution of the data points. A Bernoulli model was used as data were binary. Maximum cluster size was set to a radius of 150 km to prevent inappropriately large clusters.

### Risk factors

Risk factors for tick presence were tested using multivariable logistic regression models, applied using the *glm* function in R with ‘family = binomial’. Selected variables which met assumptions for logistic regression were first analysed using univariable logistic regression for continuous independent variables and Chi-square for categorical independent variables. Any variables with a *P*-value < 0.25 were selected for multivariable analysis [48]. The number of variables included in the initial multivariable model did not exceed the frequency of the least common outcome (presence of ticks) divided by 10 [49]. Categorical variables were dummy-coded and the reference levels were selected as those with the lowest probability of reporting ticks [50]. The final models were selected using stepwise selection to minimise the AIC (Akaike information criterion) value. The variance inflation factor (VIF) was used to check that multicollinearity between explanatory variables was low (< 4), using the *vif* function in the *car* R package (v.3.0.5; [51]). Model accuracy was assessed using the area under the receiver operating curve (AUC) (*AUROC* function in InformationValue R package (v.1.2.3; [52]) which plots sensitivity (the true positive rate) against 1 – Specificity (the false positive rate) at different threshold values. Values range from 0.5 to 1.0, with 1.0 depicting a perfect model, which would correctly detect 100% of both true and false positives. The threshold for sensitivity and specificity was selected to optimise both, by using maximum Youden’s Index (*optimalCutoff* function in InformationValue R package). Odds ratios and confidence intervals were calculated as exp(β), where β is the coefficient estimate and using profile likelihood confidence intervals (*confint* function in R), respectively, to assess the relative impact of variables in the final model on the reported presence of ticks.

## Results

### Questionnaire respondents

**Fig. 1:**
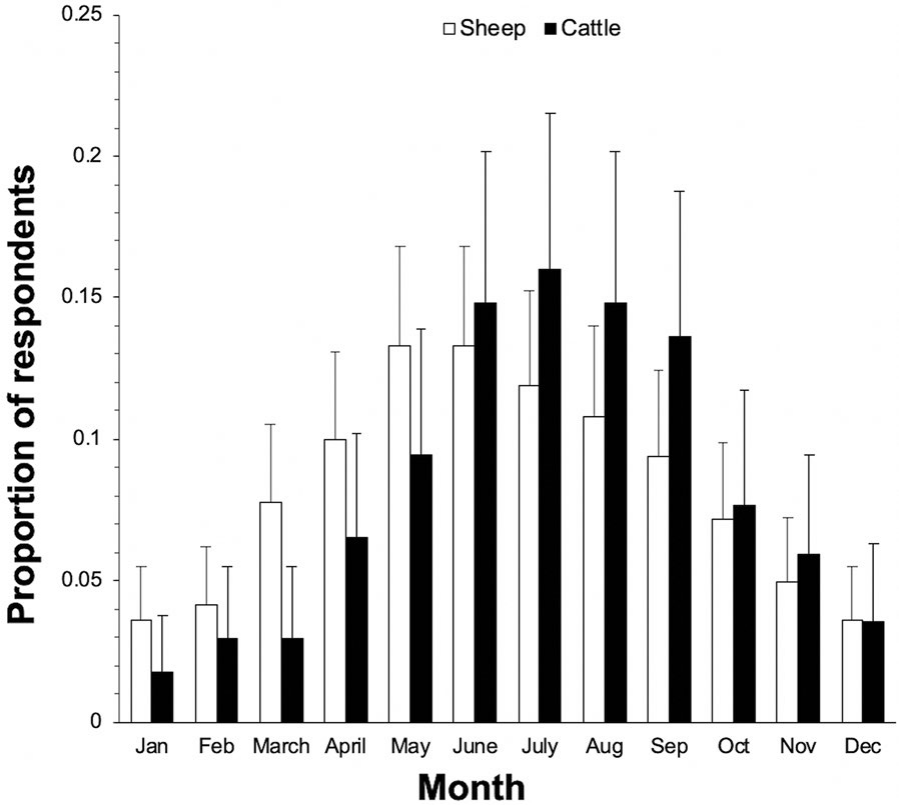
Sheep and cattle farms reporting tick infestation in each month in a retrospective questionnaire survey in Great Britain as a proportion of the number of sheep or cattle respondents (± 95% confidence intervals)

The overall questionnaire response rate was 13.4% (*n* = 964), with 926 respondents providing valid postcodes (906 of these were full postcodes, with the remaining 20 valid to at least district level). Respondent numbers were highest in the southwest of England and Wales, which is consistent with the distribution of sheep and cattle farms [38, 39]. Of the total respondents, 17% (CI ± 2; *n* = 159) farmed only sheep, 33% (± 3; *n* = 316) farmed only cattle, and 51% (± 3) (*n* = 489) farmed both. Of the farms with cattle, 72% (± 3; *n* = 580) farmed beef, 13% (± 2; *n* = 104) farmed dairy, and 15% (± 3; *n* = 121) farmed both. The majority of farms, 84% (± 2; *n* = 810), were conventional and 5% (± 1; *n* = 50) were organic, with 11% (± 2; *n* = 104) unspecified. Of the respondents, 63% (± 3; *n* = 605) described their farms as being lowland, 31% (± 3; *n* = 294) as upland, 3% (± 1; *n* = 25) as both, and 4% (± 1; *n* = 40) did not specify. The data were generally representative of the underlying holding population of England, Wales and Scotland, in terms of holding density [38, 39], ratio of dairy to beef farms [34] and ratio of upland to lowland farms [40]. Ticks were reported in all months (Fig. 1). The proportion of ticks reported each month followed a normal distribution from January to December with the highest proportion of reports during May-July. This is consistent with the unimodal peak of tick activity characteristic in environments with cold winters [10], so no questionnaires were excluded on the basis of temporal trends.

### Tick prevalence

**Fig. 2:**
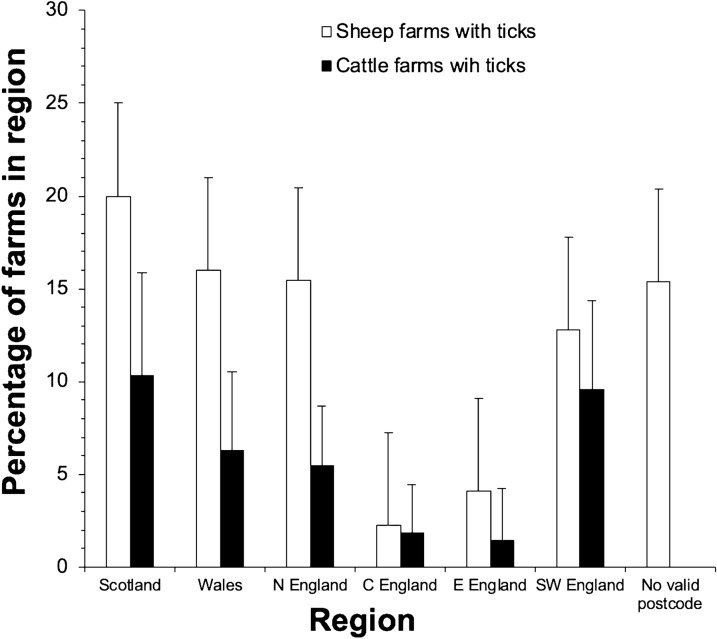
The percentage of sheep farms and cattle farms in a retrospective questionnaire survey in Great Britain reporting tick infestation relative to the number of respondents in that region (± 95% confidence intervals)

After internal validation, the total number of sheep farm respondents was 642 and the total number of cattle farm respondents was 797. The overall prevalence of farms reporting tick presence was 13.2% (CI ± 2.6; *n* = 85) for sheep farms and 6.2% (± 1.7; *n* = 49) for cattle farms. Overall, the prevalence of sheep farms with reported tick presence was higher than the prevalence of cattle farms with reported tick presence (*χ*^2^ = 18.41, *n* = 1380, *P* < 0.001). When stratified by region, the prevalence of sheep farms reporting ticks was higher than the prevalence of cattle farms reporting ticks in all regions, but this difference was only significant in Wales (*χ*^2^ = 4.93, *n* = 256, *P* < 0.05) and the north of England (*χ*^2^ = 8.97, *n* = 368, *P* < 0.01; Fig. 2).

The prevalence of farms reporting tick presence differed significantly between regions for both sheep farms (*χ*^2^ = 20.17, *n* = 648, *P* < 0.01) and cattle farms (*χ*^2^ = 15.35, *n* = 805, *P* < 0.05). The prevalence of sheep farms (as a percentage of the number of farms in each region) was highest in Scotland at 20.0% (± 8.0%, *n* = 19) and Wales at 16.0% (± 6.6%, *n* = 19) and lowest in east England at 4.1% (± 5.5, *n* = 2) and central England at 2.2% (± 3.1, *n* = 2) (Fig. 2). The prevalence of cattle farms was highest in Scotland at 10.3% (± 5.5, *n* = 12) and southwest England at 9.6% (± 4.8, *n* = 14) and lowest in east England at 1.4% (± 2.8, *n* = 1) and central England at 1.9% (± 2.65, *n* = 2) (Fig. 2).

The prevalence of farms reporting tick presence was 24.3% (± 5.3; *n* = 61) on upland sheep farms, compared to 4.3% (± 2.1; *n* =15) on lowland farms. For cattle farms, prevalence was 10.0% (± 3.8; *n* = 24) for upland farms and 3.8% (± 1.7; *n* = 19) for lowland farms.

### Spatial distribution of ticks

G function analysis showed that respondent density differed significantly from CSR, as would be expected due to the heterogeneous nature of underlying farm density. Comparison of the case/control L functions showed that case points (reported tick presence) were more clustered than control points (reported tick absence) at radii > 5 km for sheep and > 7.5 km for cattle.

**Fig. 3:**
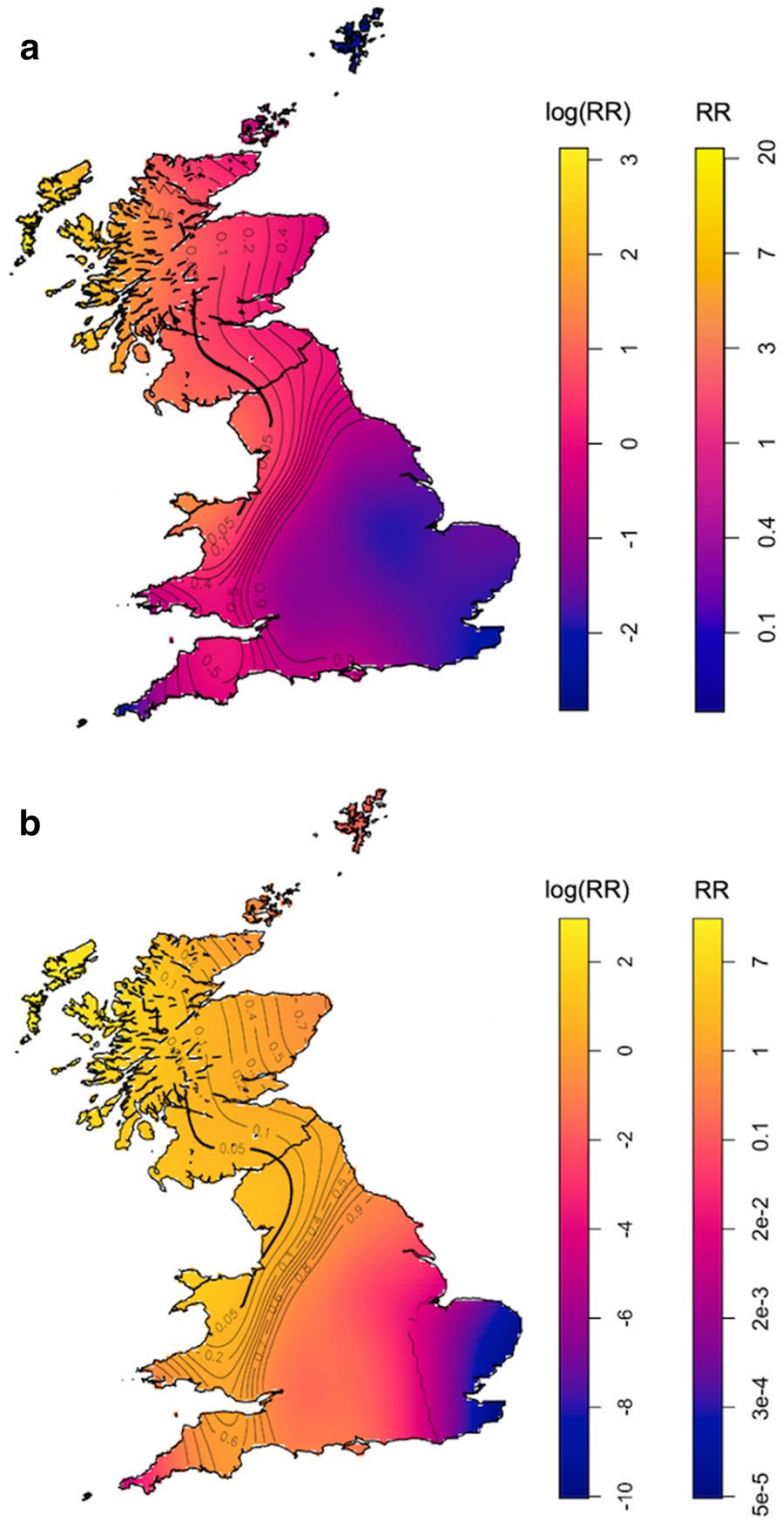
Relative risk (RR) of farms reporting tick infestation in sheep (**a**) and cattle (**b**) in a retrospective questionnaire survey in Great Britain with tolerance contour lines overlain. Lighter colours indicate higher risk and areas with significantly higher risk (*P* < 0.05) shown by the bold contour. The colour scales show log and raw relative risk

The relative risk of farmers (Fig. 3a) reporting sheep ticks and tolerance contours showed that north Wales, northwest England and western Scotland, were areas of statistically significantly elevated risk (Fig. 3a; *P* < 0.05). Cases in these areas were also confirmed as significantly clustered by SaTScan^TM^ analysis (Table 1).

**Table 1:** The location (latitude and longitude), radius (km), number of respondents, tick prevalence and relative risk for significant clusters of cases of farms with tick infestation in sheep and cattle, as identified by SaTScan^TM^ analysis of data from a retrospective questionnaire survey in Great Britain

Farm	Cluster location (lat/long of cluster centroid)	Cluster radius (km)	No. of respondents in cluster	Tick prevalence (%)	Relative risk
Sheep	N Wales*** (52.94, − 4.43)	57.23	23	65.2	5.43
	NW England*** (54.41, − 3.43)	44.45	21	66.7	5.49
	SW Scotland* (55.33, − 5.69)	70.16	5	100	7.52
	N Scotland* (56.48, − 5.98)	103.29	17	58.8	4.62
Cattle	N Wales*** (52.94, − 4.43)	57.23	24	54.2	7.79
	NW England*** (54.29, − 3.26)	46.32	29	48.3	7.03
	W Scotland** (55.48, − 5.98)	103.29	5	100	12.78

*P < 0.05, **P < 0.01, ***P < 0.001

Spatial heterogeneity in predicted relative risk was smaller for cattle ticks, but similar to the analysis for sheep farms, areas of statistically significantly elevated risk were identified in north Wales, northwest England and Scotland (Fig. 3b; Table 1). Although the reported prevalence of cattle tick cases was highest in southwest England, when considering case points on a more continuous geographical scale, taking into account the underlying distribution of respondents, cases were not found to be significantly clustered in this region.

### Risk factors for tick presence

For sheep, variables included in the initial model, based on univariable analysis were: terrain type (upland/lowland), flock size, farm type (organic/conventional) and region, but farm type was eliminated from the model during stepwise selection. After farms with missing data were removed, 480 remained in the final model. The VIF was < 4 for all variables in the final model. Significant risk factors for reported tick presence on sheep were upland terrain, larger flock sizes and being located in southwest England (Table 2; AUC = 0.77, *χ*^2^ = 480, residual deviance = 305.4 (*df* = 472), null deviance = 377.0 (*df* = 479)) (Table 2).

**Table 2:** Risk factors for tick infestation on farms, included in logistic regression models, for sheep (*n* = 480) and cattle (*n* = 711) farms based on data from a retrospective questionnaire survey in Great Britain, showing the coefficient estimate ± standard error and the odds ratio (± 95% confidence interval)

Farm	Risk factor	Coefficient estimate (± SE)	Odds ratio (95% CI)
Sheep	Terrain***		
	Lowland	–	1.00
	Upland***	1.92 (0.43)	6.84 (3.04–16.90)
	Flock size (log10)***	1.37 (0.39)	3.94 (1.88–8.80)
	Region*		
	C England	–	1.00
	Wales	0.44 (0.81)	1.56 (0.37–10.69)
	N England	1.11 (0.80)	3.03 (0.77–20.30)
	E England	1.17 (1.06)	3.22 (0.35–29.58)
	Scotland	1.02 (0.82)	2.77 (0.65–19.21)
	SW England*	2.0 (0.82)	7.13 (1.68–49.64)
Cattle	Terrain*		
	Lowland	–	1.00
	Upland*	0.89 (0.39)	2.44 (1.15–5.36)
	Livestock type**		
	Cattle only	–	1.00
	Cattle and sheep**	1.26 (0.47)	3.53 (1.51–9.69)
	Region**		
	C + E England	–	1.00
	Wales	0.99 (0.83)	2.68 (0.60–18.80)
	N England	2.61 (0.81)	2.61 (0.63–17.69)
	Scotland	1.45 (0.82)	4.26 (0.10–29.32)
	SW England**	2.27 (0.77)	9.68 (2.61–62.82)

*P < 0.05, **P < 0.01, ***P < 0.001

*Note*: ‘Upland’ refers to regions classified as Less Favoured Areas and characterized by rough grazing, heathland and moorland [55]

For cattle, variables included in the initial model, based on univariable analysis, were: terrain type (upland/lowland), livestock type (cattle only farm/cattle and sheep farm), cattle type (beef farm/dairy farm/both) and region, but cattle type was eliminated from the model during stepwise selection. After farms with missing data were removed, 711 remained in the final model. The VIF was < 4 for all variables in the final model. Significant risk factors for reported tick presence on cattle were upland terrain, presence of sheep and being located in southwest England (Table 2; AUC = 0.73, *χ*^2^ = 711, residual deviance = 285.5 (*df* = 704), null deviance = 319.1(*df* = 710)).

### Tick-borne disease (TBD)

**Fig. 4:**
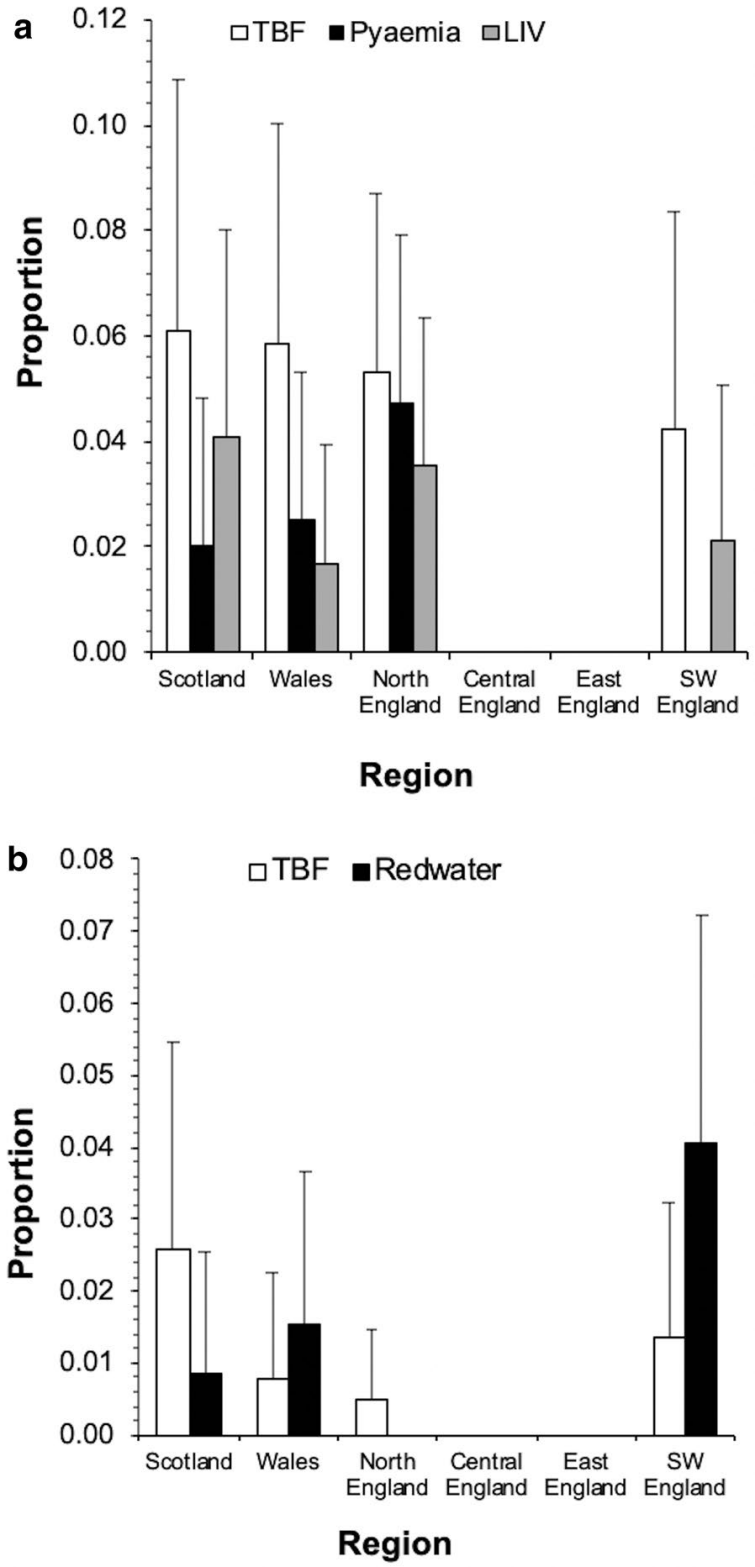
The percentage (± 95% confidence intervals) of regional farm respondents to a retrospective questionnaire survey in Great Britain reporting tick-borne disease for sheep (**a**) and cattle (**b**)

The prevalence of farms reporting at least one TBD case was 5.7% (± 1.8; *n* = 37) for sheep and 2.0% (± 1.0; *n* = 16) for cattle. Of those that reported finding ticks on their animals, 43.5% (± 10.5; *n* = 37) of sheep respondents and 32.7% (± 13.1; *n* = 16) of cattle respondents also reported having at least one TBD. Of farms reporting disease, 5.4% (± 7.3; *n* = 2) of sheep disease cases and 18.8%(± 19.1; *n* = 3) of cattle disease cases were reported to be diagnosed by a veterinarian or diagnostic laboratory. In sheep, the most common TBD was tick-borne fever (4.2 ± 1.5%; *n* = 27) (Fig. 4). In cattle, redwater was the most reported TBD (1.2 ± 0.8%; *n* = 10) (Fig. 4). The density of respondents reporting sheep disease was highest in Wales and northwest England and cattle disease in southwest England. Due to the low number of disease case points, it was generally not possibly to identify areas of significantly elevated risk; however, SaTScan^TM^ did identify a significant cluster of tick pyaemia in sheep in northwest England and of redwater in cattle in southwest England (Table 3). Of sheep farm respondents reporting disease, 97.1% (± 5.4; *n* = 34) were from upland farms. Of cattle farm respondents, 57.1% (± 24.3; *n* = 8) were from upland farms.

**Table 3:** The location (latitude and longitude), radius (km), number of respondents, disease prevalence and relative risk for significant clusters of tick-borne pyaemia cases in sheep and redwater in cattle, as identified by SaTScan^TM^ analysis of data from a retrospective questionnaire survey in Great Britain

Disease cluster	Cluster location (lat/long of cluster centroid)	Cluster radius (km)	Number in cluster	Disease prevalence (%)	Relative risk
Pyaemia in sheep	NW England* (54.29, − 3.26)	31.19	15	33.3	25.29
Redwater in cattle	SW England* (50.38, − 4.00)	139.79	109	6.4	21.35

*P < 0.05

## Discussion

The spatial analysis approach used here identifies clusters, areas which contain a higher density of points than would be expected whilst accounting for the underlying distribution of the respondents to the survey. The distribution of tick infestation and tick-borne disease prevalence in sheep and cattle reported here are consistent with the known distribution of *I. ricinus* [22, 53, 54]. Overall, 13% of sheep farms and 6% of cattle farms reported that their animals had had ticks in the study year, but with areas of significantly higher prevalence in north Wales, northwest England and western Scotland. Livestock in these regions primarily graze upland pastures and this was a significant risk factor for tick presence. The prevalence of tick infestation on upland farms was higher than the national prevalence at 24% and 10% for sheep and cattle, respectively and the prevalence of ticks on farms in statistically significant “hot spot” clusters ranged between 48–100%. Upland regions, which are classified by the EU as ‘Less Favoured Areas’ characterized by rough grazing, heathland and moorland [55], often contain a high density of questing ticks due to the combination of appropriate microclimates suitable for tick survival and abundant wildlife hosts [12, 56] and are therefore areas of high contact between livestock and ticks. Although tick populations can still be high in lowland regions, they are more limited by the lower availability of suitably humid microhabitats [57].

It is notable that for cattle the presence of sheep on the farm was a significant risk factor for tick infestation. Although deer are important hosts for ticks [25], especially in Scotland [58], sheep have been shown to maintain stable tick populations in upland regions, in the absence of other wildlife hosts, acting as hosts for all *I. ricinus* life-cycle stages [12, 59]. Hence, sheep are able to act as important maintenance hosts for tick populations in upland areas. It was suggested by Evans [60] that on mixed farms, because sheep are turned out onto pasture earlier than cattle, sheep may be a particularly important food source for the early spring population of ticks and the presence of sheep co-grazing may increase the tick population, but in some circumstances may also help to reduce the infestation on cattle.

Although under some conditions there may be a positive relationship between pathogen prevalence and tick density, this is highly variable [61] and tick presence or absence has been found to be a better predictor of pathogen transmission risk than tick abundance [62]. Therefore, risk based upon presence and absence data gives valuable information on the areas where livestock are most at risk from tick-borne disease, although presentation of clinical cases will also depend upon population immunity. Host density is also important for disease transmission, as has been found with LIV models [1] and the areas of elevated risk for tick presence are also generally areas of high livestock density [38, 39]. Although there were too few disease cases in the present study to allow relative disease risk to be mapped, the density of reported disease cases generally mirrored the density of reported tick cases. However, an exception was redwater in cattle, where a significant cluster of cases was found in southwest England. In 2006, Barton et al. [63] also found a high reported prevalence of redwater in a survey of cattle farms in the south-west, with 66% of farms reporting ticks also reporting redwater. Redwater is endemic to the UK, but clinical cases are generally only apparent when there is a breakdown in population immunity [9]. Cases may be more prevalent in the southwest because of less consistent contact between cattle and ticks, resulting in occasions where cattle are unexposed at a younger age, but are then later grazed on tick infested pastures. Further investigation of seroprevalence in cattle, the prevalance of *B. divergens* in questing ticks and the management factors that lead to a higher redwater risk in this area, is required.

Relatively high levels of variation in the number of cases of tick-borne diseases across regions has been demonstrated previously [64]. Using records of bovine babesiosis and anaplasmosis available from the Norwegian cattle and sheep health recording systems for 2006–2015, the incidences of livestock diseases were shown to be lower in eastern compared to western Norway. Climate and the much lower populations of sheep and cattle in the east were considered to contribute to this pattern [64]. In contrast, qualitative assessment of redwater cases reported in a survey of Irish farmers and veterinary practitioners found no observable foci of infection [9]. However, spatial statistics quantifying risk are necessary to elucidate spatial patterns which are not obvious based on qualitative assessment alone and correct for sampling bias [65]. The underlying distribution of respondents can vary for a number of reasons, such as sample selection bias due to differences in response rates between regions or farming sectors, or simply due to the underlying distribution of farms, which may lead to false conclusions of “hot spots” for infection in regions of high farm density based on qualitative assessment alone. The analyses applied to the data here provide robust statistical estimates of the spatial distribution of risk, taking into account the potential spatial bias of respondents through applying a presence/absence design.

When analysing risk, it is important to consider the effects of spatial scale [66]. When considered on a continuous scale in the spatial analysis, cases in southwest England were not significantly clustered, but the southwest was significantly associated with tick presence in the multivariable analysis (Table 2). The high tick prevalence in this region cannot be explained by the other factors in the models. Mapping on the relatively broad scale used here may not detect fine-grained variations in risk [66], but at this scale results are buffered against variations in microclimate which affect tick distribution on a local scale [67], allowing relative risk to be assessed in relation to broader trends, such as host density and the macroclimate [26]. Host densities and climatic variables were not directly included in the models however, so it is important to note that factors that appear as significant correlates of tick and TBD prevalence may be proxies for these more-influential drivers.

Some caution is also required with questionnaire surveys. Although they allow the collection of large data sets, they rely on accurate reporting by farmers. Reporting tick presence requires farmers to be aware of what ticks are and to be in close enough contact with livestock to spot their presence. The higher reported tick prevalence in sheep compared to cattle, for example, may be associated, to some degree, with the more frequent handling of sheep compared to beef cattle. Similarly, in terms of TBD, it is likely that farmers are under-diagnosing and may be misinterpreting clinical signs; notably, overall farmers reported that only around 11% of reported TBD cases were confirmed by a veterinarian or laboratory and two farms reported the presence of redwater in sheep, despite this not being an ovine disease. It should also be noted that this study excluded farms with relatively small numbers of animals, specifically to exclude smallholders and ‘hobby farmers’, since they might not be representative of commercial husbandry practices. However, the proportion of such holdings varies across the country, which may affect the contributions of these animals to the overall landscape prevalence of ticks and TBD. This possibility requires further investigation.

Effective control in “hot spot” regions, treating livestock with insecticides so that they act as “lethal traps”, may result in reduced tick attachment, not just to livestock, but also to other tick hosts [56, 68]. Upland farming areas represent 74% of the UK’s national parks [55], therefore the areas of elevated risk to livestock include areas of high potential contact between people and ticks. Effective control of ticks on livestock, particularly sheep in these areas, could reduce the risk of tick bites in the human population and minimise Lyme disease transmission *via* ticks co-feeding on sheep [59]. Treatment of livestock hosts has been shown to be effective in reducing disease risk to other hosts in LIV disease models, when deer populations are low [69]. However, overuse of insecticides with this strategy is also likely to hasten the selection for resistance, so alternative methods of tick control, such as the use of resistant or resilient breeds or pasture spelling, may be more appropriate [12, 31], although care should be taken if population immunity is suspected.

## Conclusions

In conclusion, tick infestation was unevenly distributed across Great Britain, with areas of significantly elevated risk in north Wales, northwest England and western Scotland. The prevalence of ticks on farms in “hotspot” clusters ranged between 48–100%. Upland farming, larger flock sizes and being located in southwest England were found to be significant risk factors for tick presence for sheep. For cattle, significant risk factors were upland farming, being located in southwest England and the presence of sheep on cattle farms. These data have important implications for assessing both the risk of tick-borne disease in livestock and optimising approaches to disease management. In particular, these data highlight the need for effective livestock tick control in upland regions and the southwest and give evidence for the importance of sheep as tick maintenance hosts in Great Britain.

## Supplementary information

**Additional file 1: Text S1.** Retrospective ectoparasite questionnaire survey sent to sheep and cattle farmers in Great Britain.

## Data Availability

The datasets generated and/or analysed during the present study are not publicly available in accordance with the data protection act, but anonymised data are available from the corresponding author upon request.
