# Supplementary material for: Distribution and prevalence of ticks and tick-borne disease on sheep and cattle farms in Great Britain
Source: Parasit Vectors. 2020 Aug 10;13:406. doi: 10.1186/s13071-020-04287-9 (PMC7419194; doi:10.1186/s13071-020-04287-9)
Supplement: Supplementary file 1 — Additional file 1: Text S1. Retrospective ectoparasite questionnaire survey sent to sheep and cattle farmers in Great Britain. [file 13071_2020_4287_MOESM1_ESM.docx]

| 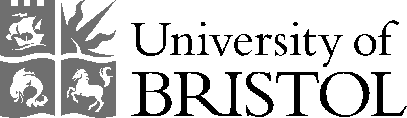**Ectoparasites of Cattle and Sheep** |
| --- |

Ticks and lice infesting cattle and sheep are becoming an increasing problem in the UK. We are conducting an anonymous survey to identify the regions and types of farms at highest risk as a first step to helping farmers and vets control these parasites more effectively. We understand that these parasites might not be a problem on your farm, but we will still be extremely grateful if you could complete and return the questionnaire; it is as important for us to understand why some farms don’t get these parasites as why some farms do.


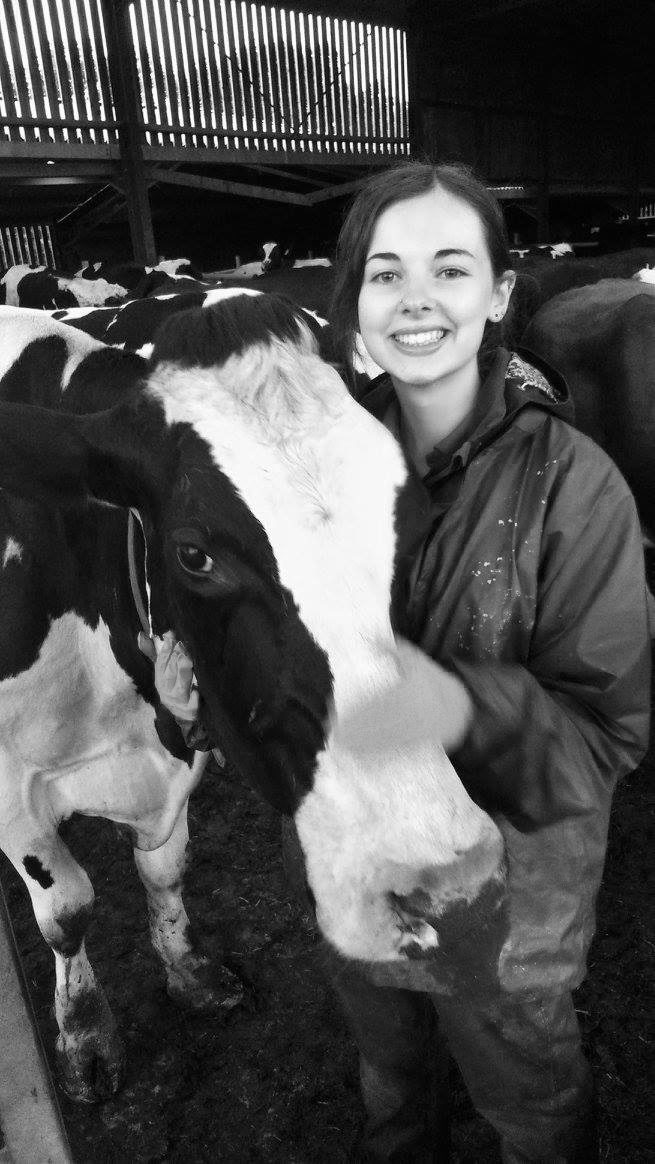


Please return your completed forms, using the freepost envelope provide, by the 12^th^ December 2018.

All information you supply will be treated as strictly confidential. Your details will not be used outside the purposes of this survey, nor will they be passed on to third parties. All data will be summarised so that no farm is individually identifiable. Please complete and return as soon as possible using the envelope provided – no stamp is needed.

Many thanks in advance for your time and help with completing this survey.

**Katie Lihou & Richard Wall**

**Veterinary Parasitology & Ecology Group, University of Bristol, BS8 1TQ, U.K**

| **About you and your farm** | |
| --- | --- |
| What is the post code of the holding where your animals are kept? | |
| Do you have: sheep , dairy cattle , beef cattle , Other …………..……………..………………… *(please specify)* | |
| Is your farm: Lowland , Upland , Hill . What is the height above sea level of your farm …….. metres? | |
| Is your farm: Organic , Conventional , Other …………………..……………………………..…………. *(please specify)* | |
| Do you use common grazing or share grazing with others?  If yes, approximately how far from the postcode given above is the common grazing? | Yes , No |

| Sheep |  |
| --- | --- |
| 1/ How many sheep did you have this year? | \|  \| Jan 2018 \| April 2018 \| June 2018 \| October 2018 \| \| --- \| --- \| --- \| --- \| --- \| \| Ewes \|  \|  \|  \|  \| \| Lambs \|  \|  \|  \|  \| \| Rams \|  \|  \|  \|  \| |
| 2/ In 2018, when did you lamb? Please circle month(s). | J F M A M J J A S O N D |
| 3/ What are the main breeds of your sheep: |  |
| 4/ In the last 12 months, have your sheep had either lice or ticks? | Yes, No  If yes, please write the approximate number of sheep affected in each month below:   \|  \| 2017 \| \| 2018 \| \| \| \| \| \| \| \| \| \| \| --- \| --- \| --- \| --- \| --- \| --- \| --- \| --- \| --- \| --- \| --- \| --- \| --- \| \|  \| Nov \| Dec \| Jan \| Feb \| Mar \| Apr \| May \| Jun \| Jul \| Aug \| Sept \| Oct \| \| Lice \|  \|  \|  \|  \|  \|  \|  \|  \|  \|  \|  \|  \| \| Ticks \|  \|  \|  \|  \|  \|  \|  \|  \|  \|  \|  \|  \| |
| 5/ Who diagnosed the infestation? | Yourself , Your Vet , Samples sent to veterinary diagnostics Lab , other |
| 6/ If you had lice or ticks, what treatment product and control method did you use? | Lice …………………………………………………………………………………………………………………………...  Ticks …………………………………………………………………………………………………………………………… |
| 7/ Did your sheep suffer from any tick-borne disease this year? | Tick-borne fever , Pyaemia , Redwater (Babesia) , Louping ill ,  Other ………………………………………………………………………………………………………………..…... |

| Cattle |  |
| --- | --- |
| 1/ How many cattle did you have this year? | \|  \| Jan 2018 \| April 2018 \| June 2018 \| October 2018 \| \| --- \| --- \| --- \| --- \| --- \| \| Dairy \|  \|  \|  \|  \| \| Beef \|  \|  \|  \|  \| \| Other \|  \|  \|  \|  \| |
| 2/ What are the main breeds of your cattle: |  |
| 3/ In the last 12 months, have your cattle had either lice or ticks? | Yes, No  If yes, please write the approximate number of cattle affected in each month below:   \|  \| 2017 \| \| 2018 \| \| \| \| \| \| \| \| \| \| \| --- \| --- \| --- \| --- \| --- \| --- \| --- \| --- \| --- \| --- \| --- \| --- \| --- \| \|  \| Nov \| Dec \| Jan \| Feb \| Mar \| Apr \| May \| Jun \| Jul \| Aug \| Sept \| Oct \| \| Lice \|  \|  \|  \|  \|  \|  \|  \|  \|  \|  \|  \|  \| \| Ticks \|  \|  \|  \|  \|  \|  \|  \|  \|  \|  \|  \|  \| |
| 4/ Who diagnosed the infestation? | Yourself , Your Vet , Samples sent to veterinary diagnostics Lab , other |
| 5/ If you had lice or ticks, what treatment product and control method did you use? | Lice …………………………………………………………………………………………………………………………...  Ticks …………………………………………………………………………………………………………………………… |
| 6/ Did your cattle suffer from any tick-borne disease this year? | Tick-borne fever , Pyaemia , Redwater (Babesia) , Louping ill ,  other …………………………………………………………………………………………………..……………… |

| **Would you be willing to help in a follow-up study by allowing us to visit your farm to collect ticks and lice from your animals?**  **If “YES”, please fill in your details:** | **No**  **Yes if yes - Name:** …………………………………….………………………………………………….  **Address:** …………………………………………………………….…………………………………………………  **Post Code:** ………………………… **Telephone:** ………………………………………………..………….  **If preferred: Email:** ……………………………………..………………………………………………………. |
| --- | --- |

If you have any questions please contact us: [bristolectosurvey2018@gmail.com](mailto:bristolectosurvey2018@gmail.com)

if you prefer, you can scan and email this survey to this address.
